# Identification of Human Embryonic Progenitor Cell Targeting Peptides Using Phage Display

**DOI:** 10.1371/journal.pone.0058200

**Published:** 2013-03-04

**Authors:** Paola A. Bignone, Rachel A. Krupa, Hal Sternberg, Walter D. Funk, Evan Y. Snyder, Michael D. West, David Larocca

**Affiliations:** 1 Mandala Biosciences LLC, San Diego, California, United States of America; 2 BioTime Inc., Alameda, California, United States of America; 3 Sanford-Burnham Medical Research Institute, La Jolla, California, United States of America; University of Cincinnati, United States of America

## Abstract

Human pluripotent stem (hPS) cells are capable of differentiation into derivatives of all three primary embryonic germ layers and can self-renew indefinitely. They therefore offer a potentially scalable source of replacement cells to treat a variety of degenerative diseases. The ability to reprogram adult cells to induced pluripotent stem (iPS) cells has now enabled the possibility of patient-specific hPS cells as a source of cells for disease modeling, drug discovery, and potentially, cell replacement therapies. While reprogramming technology has dramatically increased the availability of normal and diseased hPS cell lines for basic research, a major bottleneck is the critical unmet need for more efficient methods of deriving well-defined cell populations from hPS cells. Phage display is a powerful method for selecting affinity ligands that could be used for identifying and potentially purifying a variety of cell types derived from hPS cells. However, identification of specific progenitor cell-binding peptides using phage display may be hindered by the large cellular heterogeneity present in differentiating hPS cell populations. We therefore tested the hypothesis that peptides selected for their ability to bind a clonal cell line derived from hPS cells would bind early progenitor cell types emerging from differentiating hPS cells. The human embryonic stem (hES) cell-derived embryonic progenitor cell line, W10, was used and cell-targeting peptides were identified. Competition studies demonstrated specificity of peptide binding to the target cell surface. Efficient peptide targeted cell labeling was accomplished using multivalent peptide-quantum dot complexes as detected by fluorescence microscopy and flow cytometry. The cell-binding peptides were selective for differentiated hPS cells, had little or no binding on pluripotent cells, but preferential binding to certain embryonic progenitor cell lines and early endodermal hPS cell derivatives. Taken together these data suggest that selection of phage display libraries against a clonal progenitor stem cell population can be used to identify progenitor stem cell targeting peptides. The peptides may be useful for monitoring hPS cell differentiation and for the development of cell enrichment procedures to improve the efficiency of directed differentiation toward clinically relevant human cell types.

## Introduction

Human pluripotent stem (hPS) cells are capable of immortal proliferation *in vitro* and differentiation into derivatives of all three embryonic germ layers [1]. As a result, the isolation of hPS cells, which include human embryonic stem (hES) cells and induced pluripotent stem (iPS) cells [2], has spurred new avenues of research to evaluate their potential to provide a renewable source of human cells for basic research and as replacement cells for the treatment of injury, aging, or any one of a number of intractable degenerative diseases such as osteoarthritis, cardiovascular disease, macular degeneration, Parkinson’s and perhaps even Alzheimer’s disease [1], [2]. Reprogramming methods for creating hES-like iPS cells from somatic cells [3] have greatly expanded the number and diversity of hPS cell lines available for research. These donor-derived hPS cells are a source of patient matched cell types for disease modeling [4], drug screening [5], and the development of potential autologous cell replacement therapies [6]. However, efficient directed differentiation methods and improved cell purification technologies will be critical for deriving various cell types with sufficient purity and known identity to meet the stringent standards required for translation into routine clinical application.

Current directed differentiation methods for obtaining specific mature cell types from hPS cells are often limited by low efficiencies of reproducibly yielding the desired cell types, and even in the best outcomes, such preparations rarely exceed 30% purity [1]. One approach to increasing the yield is enrichment of desired cell types using one or more progenitor-specific markers. For example, cell enrichment using surface antigens that define progenitor populations has been used to improve the yield of the desired cell types such as neural and cardiomyocyte progenitors [7], [8]. Progenitor surface markers could also be useful for monitoring and validating hPS differentiation and for high throughput screening of reagents that stimulate differentiation toward a given lineage. However, apart from extensively mapped hematopoietic progenitor markers, there is a paucity of validated cell surface antigens for most embryonic progenitor cell lineages.

Phage display is a powerful ligand selection method that has been applied both *in vitro* and *in vivo* for the identification of cell-specific targeting peptides [9], [10]. Peptide libraries displayed on phage particles are selected by repeated rounds of enrichment for target binding phage. Displayed peptides, genetically expressed on phage coat proteins, are identified by sequencing recovered phage DNA. A distinct advantage of phage display is that it is a non-biased approach that does not require prior knowledge of the targeted cell surface receptor. However, selection against a mixed population of differentiated hPS cells is challenging because the cellular heterogeneity limits the abundance of each of the various cell type specific surface targets. Clonal expansion of cells derived from hPS cell differentiation could provide a more abundant source of progenitor cell surface targets for phage selection. Over 140 distinct clonal embryonic progenitor cell lines have been derived from hES cells using a combinatorial cell cloning approach (the ACTCellerate Initiative) that resulted in a diverse assortment of clonally pure, scalable cell lines that were selected under a variety of cell culture and differentiation conditions [11]. We reasoned that selection against clonal progenitor cell lines derived from hPS cells could be used to overcome the problems associated with selection against a heterogeneous differentiating cell population. We therefore selected a 12-mer peptide display library against one of these cell lines, W10, to test whether this approach would yield peptides capable of targeting progenitor cell subpopulations with restricted lineage potential. Negative selection was included to remove peptides that bind mature adult fibroblasts from the library prior to positive selection on W10 cells. The peptide phage library complexity was significantly reduced after the second and third rounds of selection from which we chose 4 W10 cell-binding peptides for further characterization. Specificity of the peptides was demonstrated by successful competition for peptide phage binding by synthetic peptides. The peptides exhibited selective binding to various degrees to certain embryonic progenitor cell lines but not others. The selected peptides were capable of delivering quantum dots to W10 cells and to lineage-specific cell populations of differentiating hES cells (H9) with preferential targeting to endodermal progenitors.

## Materials and Methods

### Cell Culture

The W10 and other embryonic progenitor cell lines was obtained from BioTime, Inc. (Alameda, CA) and human pluripotent stem cells (hES cell line, H9) were obtained from the Stem Cell Core at Sanford-Burnham Medical Research Institute (La Jolla, CA). Embryonic progenitor cell line (P12–30), human dermal fibroblasts (Invitrogen, P2–10) and coronary artery smooth muscle cells (CASMS) (Lonza, P6–19) were grown following manufacturers’ instructions. Human embryonic stem cells (P37–55) were cultured as colonies using standard conditions [24].

### Differentiation into Cell Lineages Representing 3 Germ Layers

For ectoderm differentiation conditions, embryoid bodies (EBs) were formed from colonies by manual techniques and grown in complete NPC media (DMEM-F12:Neurobasal media 1∶1+50 µl/ml BIT9500 Serum substutite (Stemgent) +1% GlutaMax +1% penicillin-streptomycin +1 µl/ml B27 supplement (Invitrogen) +5 mM nicotinamide +5 µg/ml insulin +20 ng/ml EGF +20 ng/ml bFGF) in low attachment plates for 6 days; before EBs were plated on fibronectin-coated wells and grown for another 3 days. For mesoderm differentiation conditions, EBs were cultured as above except that media was DMEM-F12 with GlutaMax +20% FBS +1% NEAA +1% penicillin-streptomycin, and plated on 0.1% gelatin-coated wells. For endodermal differentiation conditions, undifferentiated H9 cells were transferred from colonies growing with MEFs to Geltrex-coated wells and grown for 2 days with MEF-conditioned media +4 ng/ml bFGF. From day 2, cells were grown for another 5 days in RPMI +0.5% FBS +100 ng/ml Activin A.

### Myodifferentiation

Cells were grown as micromass cultures by plating 200,000 cells/10 µl on 0.1% gelatin-coated wells for 1.5 h before addition remaining of media. Micromass cultures were differentiated in myodifferentiation media (Smooth Muscle Cell Media 2 and Supplement Mix (PromoCell) +1% GlutaMax +1% penicillin-streptomycin +1 mM pyruvate +10 µM dexamethasone +350 µM l-proline +170 µM l-ascorbic acid +6.25 µg/ml insulin +6.25 µg/ml transferring +6.25 µg/ml selenious acid +1.25 mg/ml serum albumin +5.35 µg/ml linoleic acid) supplemented with 10 ng/ml TGFβ3.

### Gene Expression Analysis

Total RNA was extracted directly from cells growing in 6-well or 6 cm tissue culture plates using Qiagen RNeasy mini kits according to the manufacturer’s instructions. RNA concentrations were measured using a Beckman DU530 or Nanodrop spectrophotometer and RNA quality determined by denaturing agarose gel electrophoresis or an Agilent 2100 Bioanalyzer. Whole-genome expression analysis was carried out using Illumina Human Ref-8v3 BeadArrays and RNA levels for certain genes were confirmed by quantitative PCR. For Illumina BeadArrays, total RNA was linearly amplified and biotin-labeled using Illumina TotalPrep kits (Ambion), and cRNA was quality controlled using an Agilent 2100 Bioanalyzer. cRNA was hybridized to Illumina BeadChips, processed, and read using a BeadStation array reader according to the manufacturer’s instructions (Illumina). Values less than 90 relative fluorescence units (RFUs) were considered nonspecific background signal.

### Immunofluorescent Detection of Differentiation Markers

To confirm lineage commitment of cells differentiated under different conditions, cells were washed with PBS and fixed with 4% p-formaldehyde for 20 min at RT. After three washes with PBS, cells were permeabilized and blocked using 5% serum (goat or donkey, depending on primary antibody) +1% BSA +0.3% Triton X-100 in PBS for 1 h at RT. Primary antibodies were diluted in 1% BSA +0.3% Triton X-100 in PBS and incubated overnight 4°C. Antibodies and dilutions used are as follows: nestin (Abcam, Ab22035) at 1∶200, α-actinin (Sigma, A7811) at 1∶200 dilution, SOX17 (Santa Cruz, sc-17355) at 1∶400 dilution, OCT3/4 (R&D Systems, AF1759) at 1∶200 dilution, or MYH11 antibody (Biomedical Technologies Inc., BT-562) at 1∶300 dilution. After three washes with PBS, cells were incubated with secondary antibody dilutions (1∶750) in 1% BSA +0.3% Triton X-100 in PBS for 1 h at RT. Antibodies conjugated to AlexaFluor 568 were donkey anti-goat (Invitrogen, A11057), goat anti-mouse (Invitrogen, A11004) or donkey anti-rabbit (Invitrogen, A10042) depending on primary antibody. Cells were counterstained with DAPI at 0.1 µg/ml for 15 min at RT.

### Selection of Cell Binding Peptides from of a Peptide Phage Display Library

Peptide phage display library (Ph.D.-12, New England Biolabs) at 2×10^11^ pfu was adsorbed against human dermal fibroblasts (HDF) (1×10^6^ cells grown for 48 h in gelatin-coated 10 cm-dish) for 1 h on ice in W10 growth media with 2% BSA (library volume: 5 ml). The subtracted library was removed from the HDF and incubated with W10 cells plated on gelatin-coated 10 cm-dish for 2 h at 37°C, with occasional mixing. Cells were washed with washing buffer (1% BSA in PBS +0.9 mM CaCl_2_+0.73 mM MgCl_2_) using 100 times the library volume. Cells were harvested in 1 volume of dissociation buffer (PBS +1 mM EDTA), washed with 2 volumes of PBS and lysed in 1/15^th^ volume of lysis buffer (30 mM Tris pH 7.5+2 mM EDTA+protease inhibitors cocktail (cOmplete, EDTA-free Protease Inhibitor Cocktail Tablets, Roche Diagnostics) on ice for 1 hour. Cells were passed through 25G needle in 1 ml syringe and insoluble material was collected by centrifugation at 18000 g for 5 min at 4°C. Cleared lysate was transferred to a clean microcentrifuge tube and kept on ice until titration and amplification following standard protocols. In total, three round biopannings were performed using similar conditions, except that the concentration of the amplified recovered phage pool was decreased to 2×10^10^ pfu for rounds 2 and 3. The recovery of the phage pool was calculated as the ratio between the recovered phage and the input phage for each round panning.

### Sequencing of Recovered Phage

Individual phage plaques from the titration plates were grown as individual phage cultures by infection of the *E.coli* bacteria strain ER2738 (New England Biolabs). DNA was extracted using the rapid purification of sequencing templates protocol (Ph.D. Phage Display Libraries, Manual from New England Biolabs, Version 1.0, 9/09) or amplified by PCR from a peptide phage dilution using primers that hybridize outside the insert (M13KE-Ext01∶3′-TTGTCATTGTCGGCGCAACT-5′; M13KE-Ext02∶3′-GCATTCCACAGACAGCCCTCA-5′). DNA was sequenced using primer -96 gIII (3′-CCCTCATAGTTAGCGTAACG-5′). The corresponding peptide sequences were analyzed using the EMBOSS suite of bioinformatic software and their similarities were identified by ClustalW analysis. Homologous peptide sequences were identified in PepBank (http://pepbank.mgh.harvard.edu) using the Smith-Waterman search algorithm against public peptide library (201572 residues in 21672 sequences) and selecting sequences with E()<1. Homologous protein sequences were indentified in the *Homo sapiens* RefSeq protein database using Blastp (PSI-Blast, position-specific iterated BLAST with word size of 3 and Blosum62 matrix, http://blast.ncbi.nlm.nih.gov/).

### Immunofluorescent Staining of Bound Phage

The binding specificity of selected phages was determined by immunofluorescent staining of bound phage to the surface of W10 progenitor cell line. Cells were plated at 100,000 cells/well in 24 well plates and incubated overnight. Phages at 2×10^10^ pfu/well were diluted in 0.5 ml of W10 growth media supplemented with 2% BSA and incubated with live cells for 2 h at 37°C. Cells were washed as for the selection experiments and fixed by 4% paraformaldehyde for 20 min at RT. Cells were washed twice with PBS and permeabilized by ice-cold MeOH for 15 min on ice. After another two washes, cells were blocked with 5% goat serum in 2% BSA in PBS for 1 h at RT or overnight at 4°C. Cells were incubated with 1∶700 dilution of rabbit anti-Fd bacteriophage antibody (Sigma, B7786) in 2% BSA in PBS for 1 h at RT. Cells were washed with 2 ml of 1% BSA in PBS three times, and incubated with 1∶1000 dilution of goat anti-rabbit AlexaFluor568 conjugated antibody (Invitrogen, A11011) in 1% BSA in PBS for 1 h at RT. Cells were counterstained with DAPI at 0.1 µg/ml after final washes and photographed by an Olympus IX71 fluorescence microscope. Images were taken and processed using MetaMorph (version 7.5.6.0, Molecular Devices), ImageJ (version 1.45b, National Institute of Health) or Photoshop (version 9.0.2, Adobe) software.

### Binding Factor Determination

Phage binding to W10 progenitor cell line was quantified by titration. Cells were plated at 100,000 cells/well in 24 well plates and incubated overnight. Phages at 2×10^10^ pfu/well were diluted in 0.5 ml of W10 growth media supplemented with 2% BSA and incubated with live cells for 2 h at 37°C. Cells were washed as for the biopanning experiments; lysis buffer was added directed to the plated cells (100 µl per well) and incubated for at least 1 h on ice. Cells were scraped from the plate with the aid of a P200 tip, transferred to microcentrifuge tubes and lysate was cleared by centrifugation (18,000 g for 5 min at 4°C). Cleared lysates were titrated by standard protocols using sequential dilutions prepared in PBS. Lysate protein concentration was measured using the microBCA assay (Pierce) using the 96 well plate format according to manufacturer’s instructions. The relative binding factor was calculated as the ratio between the recovery (output/input) per µg of protein for the candidate phage and the M13KE control phage. Duplicate independent experiments were performed for binding factor determination.

### Peptide Competition for Phage Binding

Cells were plated at 100,000 cells/well in 24 well plates and incubated overnight. The corresponding synthetic peptide for each peptide phage or control peptides (unrelated or scrambled sequence peptide) were pre-incubated with cells at 5 nM or 5 µM in W10 growth media supplemented with 2% BSA for 30 min at 4°C. Peptide phages at 2×10^10^ pfu/well were added to the peptide dilution and incubated with live cells for 1 h at 4°C. Peptide phage bound to cells was detected by immunostaining and fluorescence microscopy using anti-phage antibody on fixed and permeabilized cells or quantified by titration of phages recovered from cell lysates. The percentage of recovered phage for the competition assay was normalized by the recovered phage in the no-peptide control. Duplicate independent experiments were performed for competition experiments.

### Cell Labeling with Peptide Targeted Qdot Complexes

2 µM of Qdot Strepavidin conjugate (Qdot605-ITK-SA, Invitrogen, Q10001MP) were diluted in 100 µl of binding buffer (supplied with Qdot605-ITK-SA) and incubated with 100-fold excess of biotinylated peptide on ice for 1 h. Uncoupled biotinylated peptide was removed from the mixture by incubating it with streptavidin magnetic beads equilibrated in PBS on ice for 30 min; placing the mixture on a magnetic stand to separate the beads and removed the complexes in solution. SA-beads were washed with PBS and combined with the recovered complexes. For control reactions, Qdots were incubated with binding buffer and treated in similar way as the peptide complexes. The concentration of Qdot-peptide complexes was estimated based on the final volume recovered. To label cells with the Qdot-complexes, 100,000 cells were plated on gelatin-coated wells of 24 well plates were incubated for 6 hours; 5 nM of Qdot-peptides in growth media was added to the cells and incubated for 16 h at 37°C. Cells were imaged after washes with PBS to remove the unbound Qdot-complexes.

### Flow Cytometry

For flow cytometric analysis of labeled cells, cells were removed from the plates using TrypLE for 5 min at 37°C. Cells were resuspended in PBS, passed through strain top tubes and analyzed using flow cytometer. Control samples included unlabeled cells and cells labeled with untargeted Qdots. For each sample, 10,000 events were quantified. The LSRFortessa flow cytometer (BD Biosciences) uses a violet laser excitation at 405 nm with a 605/23 bandpass filter to detect Qdot605 and the yellow laser excitation at 561 nm with a 670/30 bandpass filter to detect Qdot655. Cell autofluorescence was detected with the blue laser excited at 488 nm and a 510/25 bandpass filter.

## Results

### Selection of W10-binding Peptides

**Figure 1:**
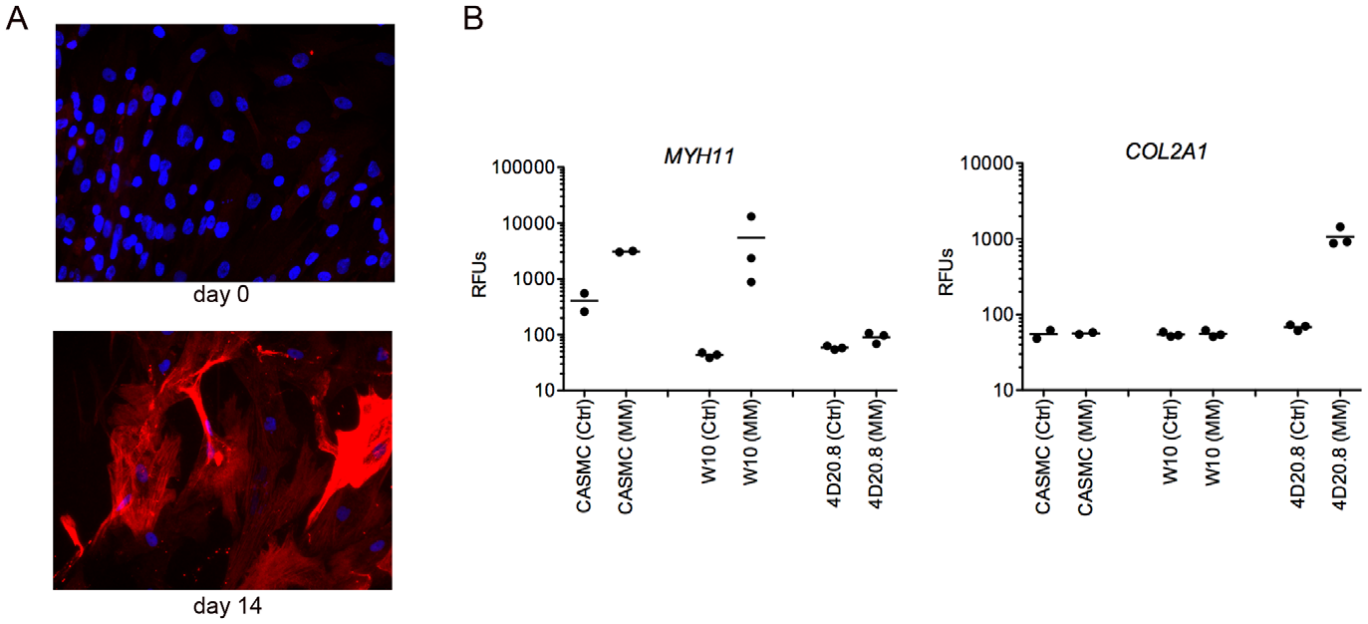
W10 is a progenitor cell line capable of smooth muscle differentiation. (A) Undifferentiated (day 0) and differentiated W10 micromass (MM) cultures in the presence of 10 ng/ml TGFβ3 (day 14). Cells were stained with anti-MYH11 antibody and DAPI. (B) W10 cells express smooth muscle marker, *MYH11,* but not cartilage marker *COL2A1* upon 14 day MM differentiation (as in A). Mean expression of the *MYH11* and *COL2A1* by Illumina microarray of day 0 undifferentiated control and day 14 differentiated MM cultures of coronary artery smooth muscle cell (CASMC), W10, and the chondrogenic cell line, 4D20.8. Values are from duplicate (CASMC) and triplicate (W10 and 4D20.8) experiments.

**Figure 2:**
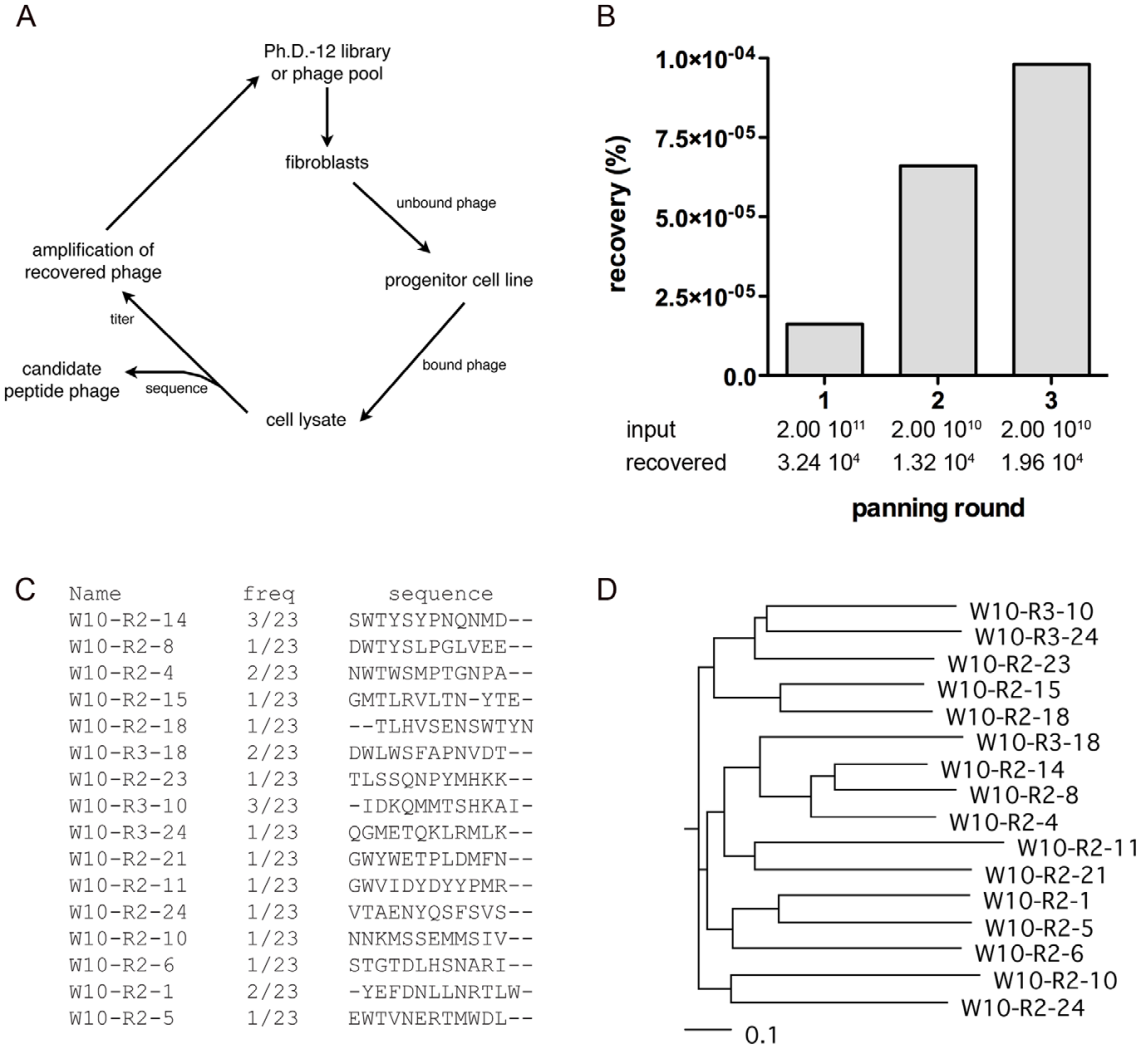
Selection of a peptide phage display library against W10 embryonic progenitor cells. (A) Peptide phages that bind to W10 embryonic progenitor cell line were enriched by 3 rounds of biopanning. PhD-12 phage display peptide library (2×10^11^ pfu, for round 1) or amplified recovered phage (2×10^10^ pfu, for rounds 2 and 3) were first adsorbed against human adult dermal fibroblasts cells and then incubated with adherent W10 cells. The phages were recovered from the cell lysate and sample phage clones were sequenced. The enriched library was amplified for further rounds of selection. (B) The percentage of input phages recovered increased with each round of selection. The percentage of input phages recovered was determined by titration of plaque forming units (pfu) in the cell lysate relative to the input pfu used for each panning round. (C) Frequency and multiple sequence alignment of peptides identified as candidate peptide phage in rounds 2 and 3 of panning generated by CLUSTAL W (2.10). (D) Phylogram based on (C) denoting peptide similarities.

The embryonic progenitor cell line, W10, expresses markers such as transcription factor, heart and neural crest derivatives-expressed 2 (*HAND2)* and distal *HOX* genes such as *HOXA4 and HOXB7.* When differentiated in high density in the presence of 10 ng/ml TGFβ3, a condition that stimulates chondrogenic differentiation of other clonal progenitor cell lines (4D20.8, SM30, 7PEND24, and E15) and the upregulation of *COL2A1* expression [12], W10 instead displays a robust differentiation to cells with markedly increased expression of smooth muscle cell markers such as smooth muscle heavy chain 11 (*MYH11)* (Figure 1), calponin 1 *(CNN1),* myosin light chain kinase *(MYLK)*, and smooth muscle actin (*ACTA2)* as measured by microarray analysis (Table S1). Cell targeting peptide phages were selected from a 12-mer linear peptide display library by 3 rounds of selection against undifferentiated W10 progenitor cells which included a negative selection against adult human dermal fibroblast (HDF) cells at each round to remove peptides binding common cell surface markers (Figure 2A). After each round of selection, the percentage of input phage recovered from the target cells increased indicating enrichment of the phage library for W10 cell binding peptides (Figure 2B). Peptide sequences were obtained from a sample of 23 individual peptide display phages recovered from rounds 2 and 3. Over-representation of several unique sequences and families of related sequences indicated a collapse of the library diversity as early as round 2. Sequences from candidate peptide phages were compared using ClustalW multiple sequence alignment software (Figure 2C). Small peptide motifs were identified in several families of related sequences (Figure 2D). The rare amino acid, tryptophan, appeared in the second position in 7 of the 24 12-mer peptide sequences suggesting selective pressure for binding to a cell surface epitope. Homology of selected peptides to known proteins can sometimes be informative for identification of candidates for the native cell binding ligand. Short peptide homologies to intracellular, membrane and extracellular proteins, identified by BLAST searching, did not indicate obvious similarities to functionally relevant domains of known cell binding proteins with the possible exception of plexin homology (Table S2A). Both W10-R2-1 and W10-R3-18 which have no homology to each other share homology in the extracellular domains of plexin B1/B3 and plexin B2 respectively, and W10-R3-18 also shares homology in the plexin binding domain of semaphorin3C (Table S2B).

### W10 Cell Binding by Selected Peptide Display Phages

**Figure 3:**
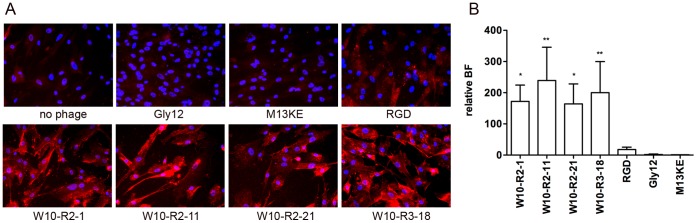
Binding of peptide display phages to W10 embryonic progenitor cell line. (A) Immunofluorescent detection of bound phages. Cells were incubated with 2×10^10^ phage particles for 2 h at 37°C; unbound phages were removed by washing and cells were fixed and permeabilized. Bound phages were detected by immunocytochemistry using rabbit anti-phage antibody and Alexa568-conjugated goat anti-rabbit antibody. Cell nuclei were stained using DAPI. (B) Quantitation of peptide phage cell binding. 2×10^10^ pfu of each candidate or controls (RGD, Gly12 and empty phage M13KE) phages were assessed for binding on 1×10^5^ W10 progenitor cells for 2 h at 37°C. Cell associated phages were recovered from cell lysates and quantified by titration. Protein in cell lysates was measured by microBCA assay. The relative binding factor (BF) is calculated as peptide phage recovery (percentage of input) relative to M13KE control phage recovery (percentage of input). Values are from triplicate experiments and shown as mean ± standard deviation. BFs for the 4 W10 peptide phage were statistical significant from the control M13KE phage (ANOVA with Dunnett’s multiple comparison tests; p values: *: <0.05 and **: <0.01). BFs for RGD and Gly12 were not statistically significant.

We analyzed 16 of the candidate peptide phages (Figure 2C) for binding to undifferentiated W10 cells using conditions similar to that used for phage library selection. The peptide phages with the strongest binding as detected by immunocytochemical staining (W10-R2-1, W10-R2-11, W10-R2-21 and W10-R3-18) are shown in Figure 3A. Little or no binding was detected for the control M13KE phage (no displayed peptide) or Gly12 control phage, which displayed a 12-mer glycine repeat peptide (Figure 3A). Immunostaining by the 4 peptide phages was stronger than a peptide phage displaying a RGD integrin binding peptide, DGARYCRGDCFDG [13]. Binding of peptide phages to W10 cells was quantified by measuring the percentage of input phages retained in the cell lysate following incubation of the cells with the phage at 37°C. The binding factor (BF) was calculated as the ratio of the percentage input recovered for each candidate phage to the percentage of input recovered using M13KE control phage. All 4 peptide phages showed similarly strong W10 binding, with BFs that were statistically different from that of the control phage M13KE; BFs for RGD and Gly12 phages were not significantly different from the M13KE control (Figure 3B).

### Peptide Specificity

**Figure 4:**
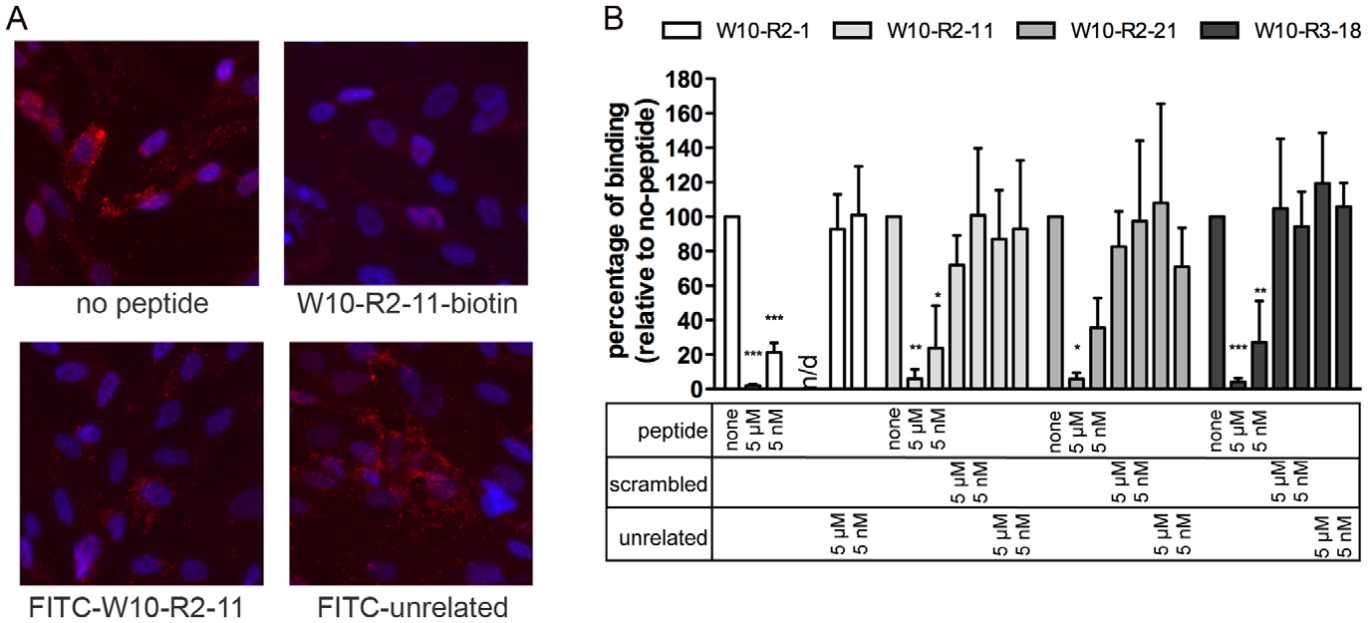
Phage binding competition with free peptide. Competition of the peptide phage with free peptide was measured using (A) Immunofluorescent detection of bound peptide phages. Chemically synthesized peptides were added to compete with binding of peptide phages to W10 progenitor cells. Cells were pre-incubated with different peptides at 100 µM or without peptide for 30 min at 4°C, followed by peptide phages (2×10^10 ^pfu) for an additional 1 h at 4°C. After washing, the bound peptide phages were detected by immunofluorescence. Peptide sequences are: W10-R2-11-biotin: GWVIDYDYYPMRGGGK(biotin); FITC-W10-R2-11: FITC-GWVIDYDYYPMRGGG and FITC-unrelated: FITC-NHVHRMHATPAY (B) Percentage of input phage recovered from cell lysate. Cells were pre-incubated with peptides at 5 µM or 5 nM, or without peptide for 30 min at 4°C, followed by peptide phages (2×10^10 ^pfu) for an additional 1h at 4°C. After washing, the recovered phage was quantified by titration. The competition is shown as percentage of no-peptide control. Values are from triplicate experiments shown as mean ± standard deviation. Competition by the corresponding free peptide was statistically significant at 5 nM and 5 µM with the exception of W10-R2-21 (only significant at 5 µM). Competition by scrambled or unrelated peptide was not statistically significant. (ANOVA with Dunnett’s multiple comparison tests; p values: *: <0.05. **: <0.01 and ***: <0.001). Peptide sequences are: peptide: X_12_GGGK(biotin); unrelated: biotin-NHVHRMHATPAY; W10-R2-11-scrambled: DYWDVGPIYRMYGGGG; W10-R2-21-scrambled: LGTMDWFWPYNEGGGG; W10-R3-18-scrambled: VSDPFDNLWTAWGGGK.

We next determined the specificity of peptide phage cell binding for the displayed peptide by performing competition experiments with synthetic peptides to indirectly measure the ability of the free peptide to bind the surface of W10 cells. The degree of phage binding following pre-incubation with free peptide was initially assessed by immunofluorescent phage staining. Competition experiments were performed at 4°C so that competition for phage binding to the cell surface could be detected in the absence of phage internalization. This resulted in a reduced phage signal compared to incubation at 37°C presumably because of the limited accumulation of internalized phage at 4°C. Representative images of W10-R2-11 surface bound phage for no peptide control and competing W10-R2-11 peptides (100 µM) are shown in Figure 4A. The N-terminal FITC-labeled peptide failed to compete with phage for binding to W10 cells. In contrast, the C-terminal biotinylated peptide successfully competed with the peptide phage. Control competition experiments with an unrelated FITC-labeled peptide indicated that the competition observed was specific. When C-terminal FITC-labeled version of the peptide was tested in similar competition experiments, the peptide was able to compete with the phage for binding to W10 cells (data not shown). These data indicate that a free N-terminus was necessary for binding to the same W10 surface molecule that is recognized by the peptide phages. We therefore performed further competition studies using C-terminal biotinylated peptides that can be linked to a variety of labeling moieties for targeted cell labeling. For these experiments the competition for peptide phage binding was quantified by measuring the percentage of input phages that were recovered from the cell lysate (Figure 4B). All 4 W10 selected peptides were able to compete with the equivalent peptide phages for binding to W10 cells. At concentrations as low as 5 nM, competition by 3 of the 4 peptides was statistically significant at p<0.05 (W10-R2-21 was the exception). Higher concentrations of competing peptide (5 µM) resulted in statistically significant competition by all 4 peptides (p<0.05). Scrambled or unrelated peptides did not compete effectively at either concentration (not statistically significant). These data indicate that cell surface binding of the 4 selected peptides was sequence specific and independent of display on the phage particle.

### Cell Labeling with Peptide Targeted Quantum Dots

**Figure 5:**
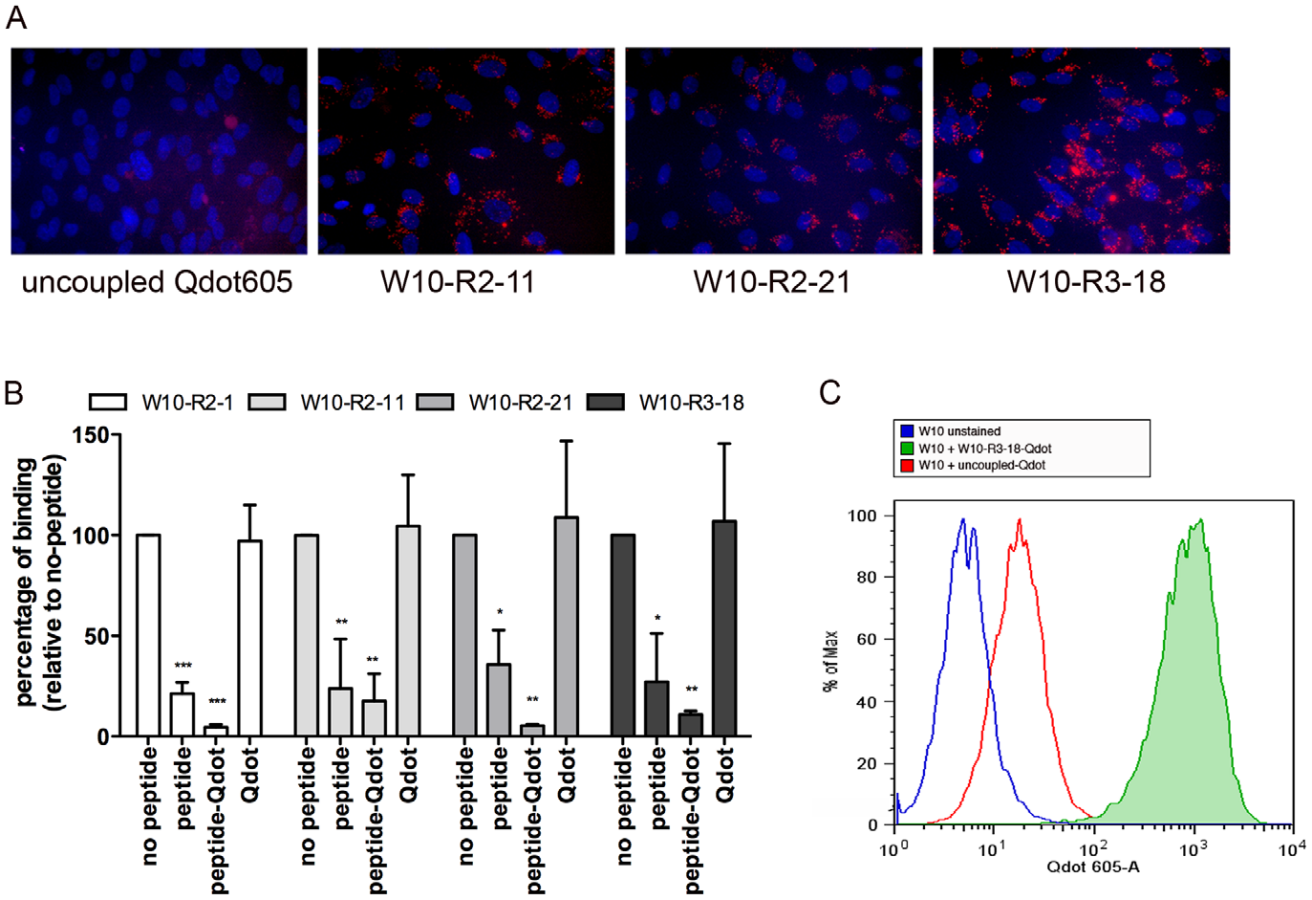
Labeling of embryonic progenitor cell line using peptide targeted Qdot605. (A) Cell targeting by fluorescent Qdots. Qdot605-ITK-SA were complexed with an excess of chemically synthesized C-terminal biotinylated peptide; unbound peptide was removed by dialysis. W10 progenitor cells were incubated for 16 h at 37°C with 5 nM of Qdot complexes, washed and imaged using a fluorescence microscope. (B) Competition with free peptide or peptide-targeted Qdots. Cells were pre-incubated with 5nM peptide, peptide targeted Qdots, or untargeted Qdots, for 30 min at 4°C, followed by addition of peptide phage (2×10^10 ^pfu) for an additional 1 h at 4°C. After washing, the recovered phage was quantified by titration. The competition is shown as percentage of no-peptide control. Values are from triplicate experiments and shown as mean ± standard deviation. Competition by corresponding free peptide or peptide-Qdot complex at 5 nM was statistically significant. Competition by uncoupled Qdots was not statistically significant (ANOVA with Dunnett’s multiple comparison tests; p values: *: <0.05. **: <0.01 and ***: <0.001) (C) Flow cytometry analysis. Cells were labeled as in (A), dissociated from the tissue culture plate using TrypLE, resuspended in PBS and analyzed in LSRFortessa flow cytometer. 10,000 events were recorded for each sample; cells were excited using the 405 nm laser and fluorescence emission was detected with the 605/12 bandpass filter. Cells labeled with W10-R3-18 peptide-Qdot complexes (green) showed higher mean fluorescent intensity than cells labeled with untargeted Qdots (red) or unlabeled W10 cells (blue).

We initially attempted to label embryonic progenitor cells using monomeric C-terminal FITC labeled peptides. However, the resulting cell labeling was minimal even at concentrations as high as 100 µM. The poor cell labeling could be due to low signal strength and/or limited internalization of the monomeric peptide because the same peptide successfully competed with peptide phage for cell binding. Accordingly, we chose to use multivalent peptide targeted Qdots to replicate both the high valency and sensitivity obtained using peptide targeted phage particles. Streptavidin conjugated CdSe-ZnS quantum dots (Qdots) were used to form complexes with the C-terminal biotinylated peptides. Qdots typically contain 5–10 streptavidin molecules bound per Qdot each of which can bind up to 4 peptides resulting in multivalent display of 20–40 peptides per Qdot. W10 cells were incubated with W10 peptide-Qdot complexes and cell labeling detected by fluorescence microscopy. Efficient cell labeling was obtained using overnight incubation at estimated concentrations of 5 nM. These conditions resulted in little or no cell labeling using untargeted Qdots (Figure 5A).

Competition experiments were used to indirectly determine the ability of the targeted Qdot complexes to bind W10 progenitor cells and to compare multivalent Qdot complexes with monomeric peptides (Figure 5B). The peptide targeted Qdot complexes successfully competed with the equivalent peptide phages for binding to W10 progenitor cells, resulting in a 80% to 95% reduction in cell binding compared to binding in the absence of competing peptide (p<0.05). Both monomer peptide and multivalent Qdot complexes competed effectively for peptide phage binding at 5 nM (>65% inhibition; p<0.05). Competition by any of the 4 peptides did not differ significantly from competition by the equivalent peptide Qdot complex (ANOVA analysis). These data indicate that differences in cell labeling between monomer peptide and multivalent Qdots may be the result of more efficient internalization by the peptide Qdot complexes rather than differences in binding. The untargeted Qdots, at the same concentration as peptide-Qdot complexes were not statistically different from the no-peptide control indicating that the peptide-Q-dot complex competition was dependent on the presence of the peptide.

**Figure 6:**
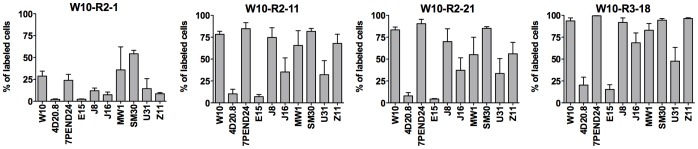
Selectivity of Qdot peptide complexes. Embryonic progenitor cell lines were labeled with Qdot complexes in their corresponding growth media and analyzed by flow cytometry as in (Figure 5C). Percentage of labeled cells was calculated by setting up gates (allowing up to 1%) using the embryonic progenitor cell line labeled with untargeted Qdots and unlabeled cells. 10,000 events were recorded for each sample. Values are from triplicate experiments and shown as mean ± standard deviation.

Cell targeted Qdots are useful reagents for labeling cells for both quantitative analysis and cell separation by flow cytometry. With this application in mind, we tested the peptide-Qdot complexes for their ability to label W10 cells for flow cytometry. Results showed a strong fluorescent shift of W10 cells labeled with W10-R3-18 Qdot complexes compared to cells labeled under similar conditions with untargeted Qdots (Figure 5C). Using flow cytometric analysis, the percentage of cells that took up the Qdot complexes was determined. Cells treated with untargeted Qdots were used for gating (Figure 5C). The percentage of W10 cells labeled with peptide-Qdot complexes ranged from 90% for W10-R3-18 to >75% for W10-R2-21 and W10-R2-11 to 30% for W10-R2-1 (Figure 6). These data were consistent with the rank order of peptide-Qdot cell labeling observed by fluorescence microscopy.

### Peptide Selectivity for Embryonic Progenitor Cell Lines

The selectivity of the peptides for W10 cells was assessed by comparing targeted Qdot labeling of W10 cells with 9 other embryonic progenitor cell lines that have been shown to be distinct cell types by genome expression profiling [11]. The percentage of cells labeled by the peptide targeted Qdots was measured by flow cytometry (Figure 6 and Figure S1). All 4 peptides showed some degree of selective cell targeting. The W10-R3-18 peptide, which was most efficient labeling peptide for W10 cells, was the most promiscuous cell targeting peptide. It bound to a high percentage of cells in 7 out of the 10 embryonic progenitor cell lines. The selective labeling profiles of W10-R2-11 and W10-R2-21 peptides were very similar to each other, with very little labeling of 2 clonal progenitor lines, 4D20.8 and E15, and a high percentage binding of the 7PEND24 cell line. Indeed, these 2 peptides share sequence identity at positions 1 and 2 (G, W), have strongly conserved residues at position 5 and 11 (D/E, M/F) and weakly conserved residues in the last position (R/N). Competition experiments showed that these 2 peptides can compete with each other for W10 cell binding (not shown). Taken together, these data suggest that the 2 peptides might bind the same cell surface epitope. More restricted cell labeling was observed with W10-R2-1 Qdot complexes. Of the 10 clonal progenitor lines tested, only W10, 7PEND24, SM30 and MW1 cell lines showed more than 15% cell labeling and no complex uptake was observed for E15 and 4D20.8 cells. The labeling of different embryonic progenitor cell lines gave an indication of the selectivity of the peptides complexes. While the binding is not exclusive to the W10 cell line they were selected on, there was a difference in the pattern of progenitor cell line targeting depending on the peptide sequence.

### Peptide Selectivity for Differentiating Pluripotent Stem Cells

**Figure 7:**
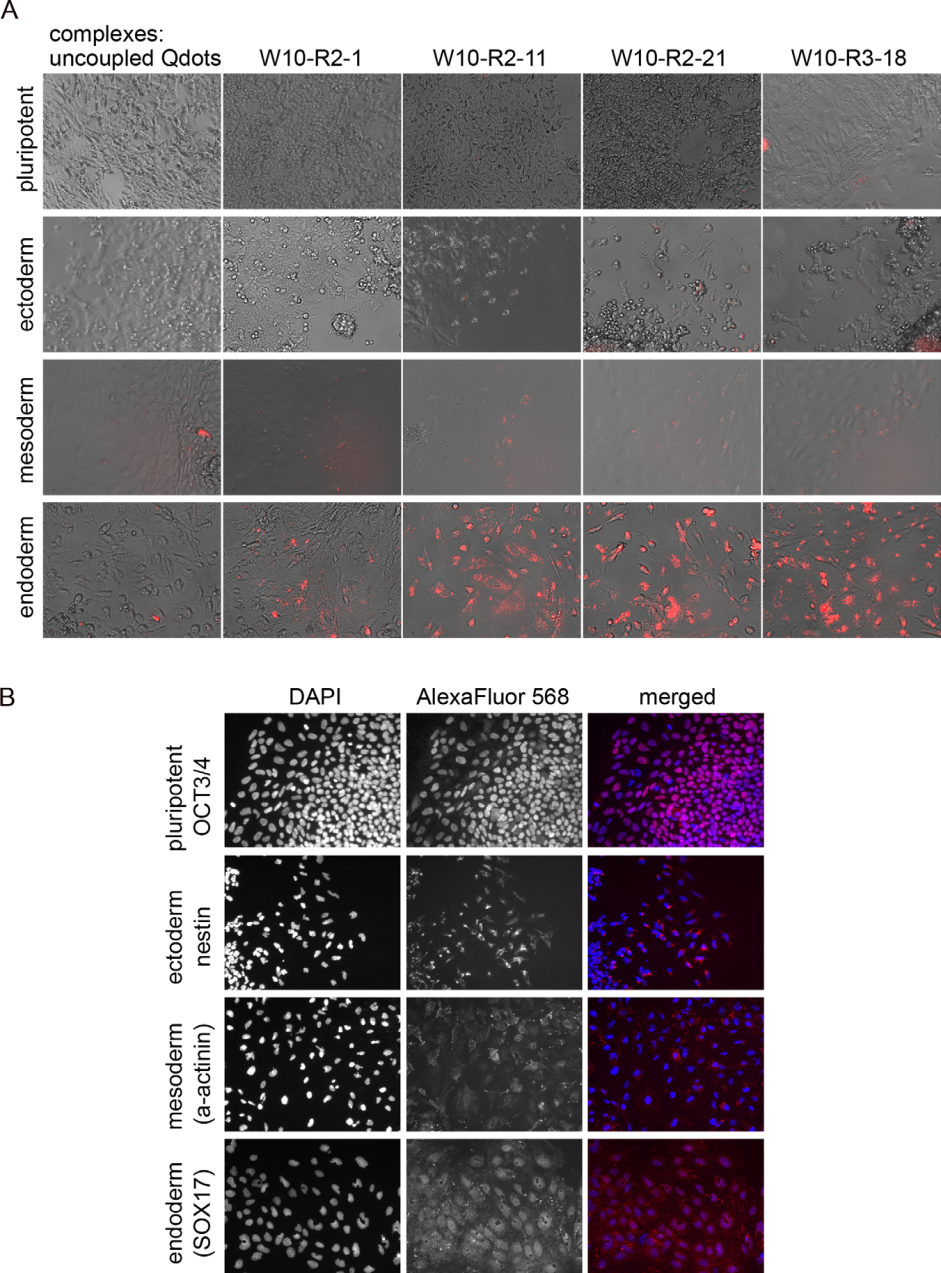
Labeling of a differentiating pluripotent stem cells using peptide targeted Qdots. (A) Selective peptide targeting of human embryonic stem cells (H9) that were differentiated into the three germ layers (see methods for protocols) for 6–8 days compared to undifferentiated control. Differentiated cells were incubated for 16 h at 37°C with 5 nM of Qdot complexes, washed and imaged using a fluorescence microscope. Representative bright-field and fluorescent signal of W10 peptide Qdot complexes (red) are shown. (B) Cells were stained with the following differentiation markers to verify the germ layer commitment: nestin for ectoderm, α-actinin for mesoderm, SOX17 for endoderm conditions and OCT3/4 for undifferentiated cells.

We next tested the W10 cell selected peptides for selective targeting of embryonic progenitors that appear in early differentiating hPS cultures under 3 different growth conditions that promote differentiation toward ectoderm, mesoderm or definitive endoderm (Figure 7). Importantly, W10-peptide complexes did not label undifferentiated H9 cells indicating that they are indeed selective for differentiated cells. Little or no cell labeling was observed when hPS cells were grown under ectoderm differentiation conditions for any of the 4 different peptide-Qdots complexes. Cell labeling was highly restricted, resulting in a few small patches of labeled cells, for hPS cells grown under mesoderm differentiation conditions and incubated with W10-R2-11, W10-R2-21 and W10-R3-18 targeted Qdot complexes but no labeling was observed with W10-R2-1 (Figure 7A). In contrast, a high percentage of cells were labeled when hPS cells were differentiated using culture conditions for definitive endoderm (high activin A, low serum) and incubated with W10-R2-11, W10-R2-21 and W10-R3-18 targeted Qdot complexes. In contrast, cell labeling was highly restricted to a small percentage of cells when the endoderm differentiated cells were incubated with W10-R2-1 complexes. The hPS cells that were differentiated under the same 3 conditions were not labeled by incubation with untargeted Qdots indicating that in each case the cell labeling was dependent on the targeting peptide (Figure 7A). Immunostaining with differentiation specific markers was used to confirm differentiation toward the appropriate lineage fate (Figure 7B). Taken together, these data indicate that the W10 selected cell targeting peptides are capable of distinguishing between different types of embryonic progenitor cells with a marked preference for targeting early definitive endodermal progenitor cells.

## Discussion

Previous studies have shown that phage display is useful for identifying peptides that target undifferentiated [14], [15], [16], differentiated cells [15] or cancer cell lines [17], [18]. We describe here a novel phage display strategy that uses selection against a clonally pure pluripotent stem cell derivative to identify peptides that selectively target early hPS cell derived progenitor stem cell populations. The peptides developed here clearly bind to one or more developmentally regulated surface markers that are absent on undifferentiated pluripotent stem cells but are detected predominantly on definitive endodermal progenitors derived from 6–8 day differentiating hPS cells. Peptide targeting to definitive endoderm was unexpected given that the peptides were selected on the W10 cell line which expresses smooth muscle and other mesodermal markers [11]. However, the targets for the cell binding peptides although restricted may be present on more than one progenitor cell type. Analysis of 10 ACTCellerate cell lines revealed highly prevalent peptide binding to multiple distinct progenitor cell lines. Therefore, a combination of cell targeting peptides may be needed to more narrowly define and isolate a particular progenitor cell type. Selection of peptide libraries on additional clonal progenitor cell lines such as those identified by the ACTCellerate initiative [11] may be useful in this regard for identifying additional progenitor targeting peptides. Further studies are needed to identify and characterize the cell surface molecules targeted by the W10 selected peptides. Once known, it will be possible to develop additional peptides and antibodies against the receptor to determine its temporal and spatial expression pattern and to better understand its role during differentiation of hPS cells.

We demonstrated specificity of the W10 selected peptides by competition experiments with the free peptides. The competition experiments demonstrated that free peptide could compete for peptide phage binding at concentrations as low as 5 nM indicating that the targeting peptides have high affinity for their cognate cell surface antigens. The lack of competition with scrambled peptides indicated that the binding for W10-R2-11, W10-R2-21 and W10-R3-18 peptides is sequence specific and not a result of non-specific interactions. Failure of the N-terminal FITC labeled peptides to compete for peptide phage binding indicated the need to replicate the free N-terminus of the peptides that is present when the peptides are displayed as fusion to the phage pIII coat protein. However, we were unable to label cells with C-terminal FITC labeled monomer peptides. We therefore developed peptide targeted Qdots for targeted cell labeling. The effective targeted cell labeling by Qdots and not monomeric peptide was likely due to the increased signal intensity and lack of quenching that is intrinsic to Qdots and the multimeric display of the peptide on the Qdots. Replicating the multimeric display of the phage particle is known to increase avidity and may increase internalization by triggering dimerization/oligomerization of the cell surface receptor which would allow for increased signal as a result of accumulation of Qdots within the cells. The vesicular pattern of Qdots and detection of cell labeling by flow cytometry following trypsinization to a single cell suspension suggests that the peptide targeted Qdots were internalized.

Several reports have shown that functionalized Qdots do not cause any deleterious effects on cell survival in vitro [19], [20] and that the delivery of Qdots by electroporation or lipofection does not disrupt early stages of mammalian development or early embryogenesis nor adversely affect embryonic stem cell viability, proliferation or differentiation [21]. Here, we have demonstrated selective cell labeling using peptide targeted Qdots and determined the percentage of labeling of live cells by flow cytometry. The peptide Qdot cell labeling was not exclusive to W10 cells but was shared to various degrees with other progenitor cell lines with little or no cell labeling of 2 lines (E15 and 4D20.8). Interestingly, these 2 cell lines share a common derivation pathway that is distinct from the other lines [11]. These data indicate that the targeting of W10 selected peptide was restricted to certain progenitor cell types but was not limited the smooth muscle progenitor cell line. Further studies are needed to determine the cell fate of progenitor cells that are targeted by the peptides. In this regard, a significant advantage of identifying peptides that can target Qdots to live cells is that they could potentially be used for labeling and isolating viable hPS derived differentiating stem cells for further culture and characterization. For example, this approach could be used to characterize the small fraction of hPS derived mesodermal cells that were labeled by peptide targeted Qdots. The persistence of the Qdot signal could also be used for progenitor cell tracking during differentiation of hPS cells to determine cell fate. The peptides could also be used to target magnetic particles as an alternative approach for separating cells using magnetic activated cells sorting which has been used successfully for preclinical and clinical cell transplant applications [22].

Cellular heterogeneity in hPS derived cell populations is a major bottleneck for the successful development of hPS derived cells for transplantation. Contamination of differentiated cells with residual pluripotent cells is of particular concern for safety because of their ability to form teratomas in vivo [23]. Cell purity is also important for consistency of non-clinical applications such as disease modeling, drug screening and drug safety testing. We have begun to address this issue by developing targeting peptides that can identify subsets of progenitor cell types for use in cell enrichment and cell exclusion procedures. The advantage of such cell enrichment steps is clearly demonstrated by over a decade of the clinical application of cell surface targeted enrichment of hematopoietic stem cells for stem cell transplants as a cancer treatment [22]. In the present study we isolated human embryonic progenitor stem cell targeting peptides that recognize certain hPS derived progenitor stem cell lines as well as hPS derived early definitive endoderm. The endodermal progenitor targeting peptides might be useful for enriching or excluding endodermal progenitors during directed differentiation. The peptide targeted Qdots could also be used to rapidly assess hPS cell differentiation capacity and to screen for reagents that direct differentiation toward definitive endoderm. Identification of additional progenitor stem cell targeting peptides using the approach described here may make it possible to improve recovery of clinically relevant progenitor cell types. This would be particularly useful for deriving patient-specific progenitors from the patient’s own reprogrammed iPS cells. For example, a recent preclinical study of one of the ACTCellerate clonal cell lines, 4D20.8, has demonstrated the ability of this cell line to differentiate to chondrocytes capable of cartilage repair in a rat knee model [12]. It may be feasible to use the phage display approach described here to isolate stem cell targeting peptides that would facilitate retrieval of the equivalent cells from patient derived iPS cells to provide a source of genetically matched stem cells for cell replacement therapy.

## Supporting Information

Figure S1 **Selectivity of Qdot peptide complexes.** (A) Fluorescence microscopy images of confluent embryonic progenitor cell lines labeled with W10-peptide Qdot complexes, showing only signal from Qdot655 channel only. (B) Overlap histograms of flow cytometric quantification of labeled cells from (A). W10 peptide Qdot complex is shown in red while control samples of uncoupled Qdots and unstained cells are shown in black and grey, respectively. Results are representative of three independent experiments.
(TIF)

Table S1 **Differentiation of W10 cell line.** Gene expression analysis of cultured coronary artery smooth muscle, W10, and 4D20.8 cells in the undifferentiated state and micromass (MM) differentiation conditions. (A) Comparative microarray relative fluorescence units (RFU) values for coronary artery smooth muscle cells, W10, and 4D20.8 in control conditions of five-day quiescence and 14 days of micromass culture. (B) Values from selected genes are compiled from data in (A), and the corresponding graphs showed the upregulation of smooth muscle heavy chain 11 (*MYH11),* calponin 1 *(CNN1),* myosin light chain kinase *(MYLK)*, and smooth muscle actin (*ACTA2)* in W10 and CASMC cells but not in 4D20.8 cells under myodifferentiation conditions.
(XLSB)

Table S2 **Analysis of binding W10 peptide phage sequences.** A) Best score hit for homologous protein sequences were identified in the *Homo sapiens* RefSeq protein database using Blastp (PSI-Blast, position-specific iterated BLAST with word size of 3 and Blosum62 matrix, http://blast.ncbi.nlm.nih.gov/). B) Sequence homology of the W10 binding peptides with plexins and semaphorin. Identical amino acids are in bold, highly similar are grey.
(PDF)
