# Supplementary figures and images for: Identification of Human Embryonic Progenitor Cell Targeting Peptides Using Phage Display
Source: PLoS One. 2013 Mar 4;8(3):e58200. doi: 10.1371/journal.pone.0058200 (PMC3587414; doi:10.1371/journal.pone.0058200)

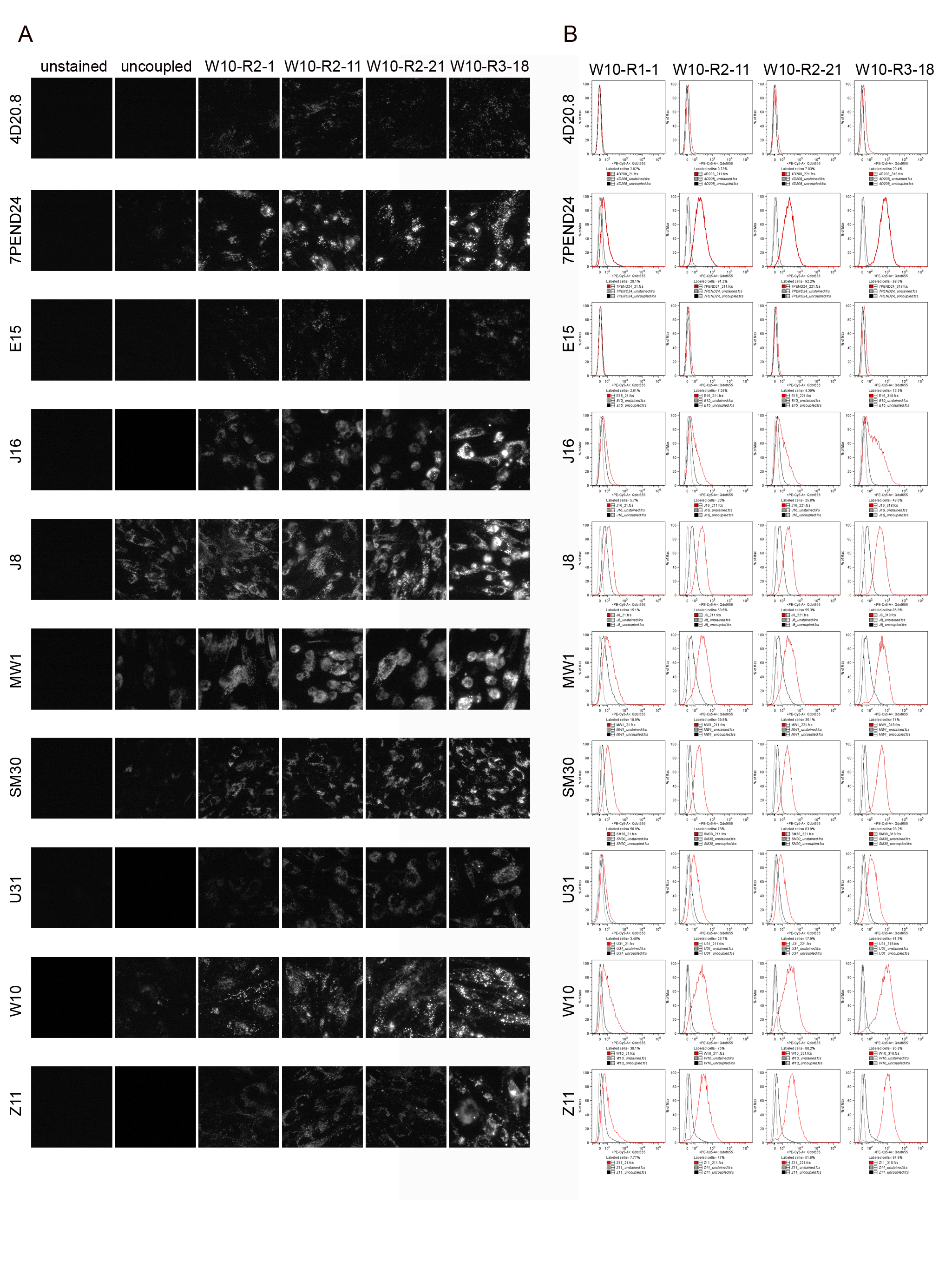

Supplement: Figure S1 — Selectivity of Qdot peptide complexes. (A) Fluorescence microscopy images of confluent embryonic progenitor cell lines labeled with W10-peptide Qdot complexes, showing only signal from Qdot655 channel only. (B) Overlap histograms of flow cytometric quantification of labeled cells from (A). W10 peptide Qdot complex is shown in red while control samples of uncoupled Qdots and unstained cells are shown in black and grey, respectively. Results are representative of three independent experiments. (TIF) [file pone.0058200.s001.tif]
