# Supplementary material for: Identification of Human Embryonic Progenitor Cell Targeting Peptides Using Phage Display
Source: PLoS One. 2013 Mar 4;8(3):e58200. doi: 10.1371/journal.pone.0058200 (PMC3587414; doi:10.1371/journal.pone.0058200)
Supplement: Table S2 — Analysis of binding W10 peptide phage sequences. A) Best score hit for homologous protein sequences were identified in the Homo sapiens RefSeq protein database using Blastp (PSI-Blast, position-specific iterated BLAST with word size of 3 and Blosum62 matrix, http://blast.ncbi.nlm.nih.gov/). B) Sequence homology of the W10 binding peptides with plexins and semaphorin. Identical amino acids are in bold, highly similar are grey. (PDF) [file pone.0058200.s003.pdf]

Supplemental Table 2. Analysis of binding W10 peptide phages.

A) PSI-Blast search on *Homo Sapiens* RefSeq protein database

name	sequences	homology	Score	aa overlap	% Identity	% Similarity (% Gaps)	Protein	Accession number
W10-R3-18	DWLWSFAPNVDT	523 WLWSFQP	25.2	7	86	86 (0)	Plexin-B1 precursor	NP_002664.2
W10-R2-1	YEFDNLLNRTLW	215 DFQHLLNRTL	25.7	10	70	80 (0)	disintegrin and metalloproteinase domain-containing protein 15 isoform 9 preprotein (and other isoforms)	NP_001248395.1
W10-R2-21	GWYWETPLDMFN	121 ETPLDM	24.0	6	100	100 (0)	palmitoyltransferase ZDHHC13 isoform 1 (also isoform 2)	NP_001001483.1
W10-R2-11	GWVIDYDYYPMR	803 GWVIYKDYQYY	28.2	11	73	73 (18)	Macrophage mannose receptor 1 precursor	NP_002429.1

B) Homology of binding W10 peptides.

protein	start	sequence	end
W10-R2-11	1	G**W**VIDYDYYPMR	12
W10-R2-21	1	G**W**Y**W**ETPLDMFN	12
W10-R3-18	1	**DWLWSF**A**PNV**D**T**	12
WNT5A-like	87	**DWLW**Y	90
Plexin B3	538	**WLWS**	542
Plexin B1	523	**WLWSF**Q**P**	529
Semaphorin 3C	19	**SF**N**PNV**N**T**	26

protein	start	sequence	end
W10-R2-1	1	**Y**E**F**D**NLLNRTL**W	12
Plexin B2	1320	**Y**Q**F NLLN**	1327
Plexin B2	190	**LL**D**RT**	194
Plexin B2	245	**NRTL**	248
